# Commentary: A “Source” of Error: Computer Code, Criminal Defendants, and the Constitution

**DOI:** 10.3389/fgene.2017.00033

**Published:** 2017-03-15

**Authors:** Duncan A. Taylor, Jo-Anne Bright, John Buckleton

**Affiliations:** ^1^Forensic Science South AustraliaAdelaide, SA, Australia; ^2^School of Biological Sciences, Flinders UniversityAdelaide, SA, Australia; ^3^Institute of Environmental Science and ResearchAuckland, New Zealand

**Keywords:** source code, STRmix, DNA profile interpretation, closed source, court challenge

Chessman (2017) warns of the current trend to admit into court unchallenged the results of complex computerized calculations. He provides a number of examples and arguments claimed to demonstrate the need for open source software to remove the “black box” element. We agree with parts of this sentiment, and the topic of this special issue, that there is a danger with those using and receiving information from black box systems.

Some care however is needed with simple diagnoses and prescriptions such as these.

Modern probabilistic genotyping software are replacing methods previously applied manually. We have great confidence in the forensic community with regard to both integrity and dedication. The previously applied processes are usually a composite of standard operating procedure and human judgment. The difference between these and probabilistic software is largely that the processes in the software are encoded.

Many disciplines are sufficiently broad that practitioners need to rely, in part, on the work of others. This is not new (for a discussion on this point see Taylor, 2016). The risk to which Chessman refers arises when the individual using the system has so little understanding that they do not know how to use the system, or when it has not worked^1^. Chessman provides some helpful suggestions for how breaking down black box barriers can be addressed on an individual and systemic scale. As developers of expert system STRmix™^2^(Taylor et al., 2013), we wish to address some of the alarmist points in Chessman (and echoed by others^3^) that gives the impression that producers of expert systems are all either incompetent or corrupt.

We first wish to correct a couple of points in (Chessman, 2017). Regarding the “erroneous assumption” referenced by footnotes 49–51: This miscode, and indeed any miscode found that has been identified in STRmix™ development or use, was identified by examination of the program's output and not the source code. It would be nearly impossible to identify subtle errors in code by viewing the code. The identification has always been a result of comparison of the results produced by a program to some known control^4^. The results of these comparisons then trigger the examination of a specific section of the code in order to discover the source of the discrepancy. Even as developers, during the developmental validation of new versions of STRmix™, we utilize the extended outputs of the software to validate, and do not validate by examination of code. A further reference (footnote 98) makes the same incorrect assumption that it was code review that lead to the discovery of a programming error. Our experience has been that even more crucial than a review of source code, is the ability to have access to outputs that demonstrate each step of a calculation. We should also note that our ongoing evaluation and testing of the software is a marker of continuous validation and refinement, rather than just fixing “errors” and “blunders.”

The second point we wish to make is that the type and magnitude of miscodes are important to consider. The majority of programming errors will lead to instances of a program “crashing” or failing to produce an answer. These types of errors are arguably inconsequential as they will not lead to any erroneous results being produced. More serious are miscodes where no errors are identified or displayed by the software. These can be split into those that will be clearly identifiable^5^ and those that are more subtle and may go initially unnoticed. Even in this latter category, the question should be asked “What effect does this error have?” If the magnitude of the difference in the result caused by the miscode is small compared with the natural variability in the results being produced^6^ then arguably the consequences are minimal. We are by no means suggesting that that these types of errors are acceptable, they should be rectified as soon as found. We simply suggest that they tend to be used for scaremongering in a manner disproportionate to their impact. Case in point is the oft quoted article (David Murray, 2015), which contains the never quoted sentence “*The DNA likelihood ratios in both the new and original statements appear to be the same*.”

We agree with the suggestion of Chessman that source code should be available for scrutiny. STRmix™ abides by one of the mechanisms that Chessman suggests, namely the ability for code to be disclosed under confidentiality agreements^7^. We note that running of STRmix™ is just the final step in a long journey of computerized activities that ultimately lead to an answer. A true challenge of all steps in the process would require the examination of the source code underlying the Java programming language in which STRmix™ is written, the Windows™ operating system on which it is run, the software used to process the raw electrophoretic data, the software used to collect the raw electrophoretic data from the electrophoresis instrument, the code used to run the electrophoresis instrument, the PCR thermocycler, the quantification instrument and a myriad of no doubt thousands of blocks of code that sit within the numerous Peripheral Interface Controllers that control hardware components.

With the advent of complex computerized evaluation of evidence, there is a shift from a time where an expert can testify to all aspects of the evaluation, to one where, at some level, the workings of an expert system are accepted without absolute understanding. This may initially seem frightening, but an examination of the bigger picture suggests otherwise. It would be difficult to argue that the use of computerized breathalyzers is a backwards step from the reliability of the Field Sobriety Test. Similarly, virtually all senior advisory bodies relating to DNA profile evaluation recognize the clear benefits of the probabilistic interpretation systems (which by nature of their complexity require computerized implementation) over the preceding manual or binary interpretation methods (Coble et al., 2015; SWGDAM, 2015). In our efforts to ensure that software is not the “source” of errors, it is important to recognize that even with the noted occurrences of these errors, the current computerized solutions, when used by trained experts, represent a vast improvement to the quality and reliability of evidence presented in court.

## Author contributions

All authors contributed to the discussions and writing of the manuscript. Points of view in this document are those of the authors and do not necessarily represent the official position or policies of the author's organizations.

## Funding

Funding to write this manuscript was provided by the author's institutions only in the sense of allowing work time to be used to develop the document.

### Conflict of interest statement

Authors are technical developers of commercial software STRmix™ but do not benefit financially from STRmix™.

## Footnotes

^1^Note that this is not an issue with just computer programs, recent history has numerous examples within forensic biology showing that a misunderstanding of the way a system works at a fundamental level can cause issues even when the calculations themselves are relatively simple and able to be done by hand (Budowle and Bieber, 2015).

^2^An expert system that analyses STR DNA profile data.

^3^For example see EPIC (https://epic.org/state-policy/foia/dna-software/).

^4^Commonly a “by-hand” recreation of the expected value(s).

^5^Such as value of a probability greater than one, or a negative amount of some substance.

^6^This may either be in the raw results due to inherent variability in the laboratory process or it may be variability in the statistical result due to an evaluation method that utilizes random number generation (Bright et al., 2015).

^7^The code of STRmix™ has been viewed under such conditions in the past.
